# “They don’t Know Better than I do”: People Prefer Seeing for Themselves Over Using the Wisdom of Crowds in Perceptual Decision Making

**DOI:** 10.5334/joc.173

**Published:** 2021-06-21

**Authors:** Merav Yonah, Yoav Kessler

**Affiliations:** 1 Department of Psychology and Zlotowski Center for Neuroscience, Ben-Gurion University of the Negev, Beer-Sheva, 8410501, Israel

**Keywords:** wisdom of crowds, overconfidence, decision making

## Abstract

Establishing the way people decide to use or avoid information when making a decision is of great theoretical and applied interest. In particular, the “big data revolution” enables decision-makers to harness the wisdom of crowds (WoC) toward reaching better decisions. The WoC is a well-documented phenomenon that highlights the potential superiority of collective wisdom over that of an individual. However, individuals may fail to utilize the power of collective wisdom as a means for optimizing decision outcomes. Using a random dot motion task, the present study examined situations in which decision-makers must choose between relying on their personal information or relying on the WoC in their decision. Although the latter was always the reward-maximizing choice, a substantial part of the participants chose to rely on their own observation and also advised others to do so. This choice tendency was associated with higher confidence, but not with better task performance, and hence reflects overconfidence. Acknowledging and understanding this decision bias may help mitigating it in applied settings.

## 1. Introduction

The finding that a group of laypeople pooling their judgments can outperform even the judgment of experts is referred to as the wisdom of crowds (WoC). Many studies have demonstrated numerous situations in which aggregated performance is superior to that of individuals. These include quantity estimation (Galton, 1907; Treynor, 1987), problem-solving (Laughlin et al., 2006), and medical diagnostics (Kurvers et al., 2016). Utilizing knowledge and opinions of collectives can potentially revolutionize a wide range of domains and lead to better, more accurate decisions. But as human decision-making is far from a normative rational process, it is possible that individuals fail to appreciate the power of collective wisdom and dismiss its valuable benefit. The present study employs a scenario where the crowd is always superior to the individual. Focusing on this type of situation, we wish to examine whether individuals are willing to rely on the WoC in their individual decisions and explore the internal and situational correlates of such behavior.

Studies from the advice-taking literature thoroughly described when, how, and how well individuals utilize the advice of others (For a review, see Bonaccio & Dalal, 2006). While advice-taking behavior depends on the characteristics of the source, the recipient, and the nature of the decision, it appears that more often than not people take advice in a suboptimal manner. Specifically, several studies suggest that while taking advice, people tend to favor their initial judgments and underweight others’ (Harvey & Fischer, 1997; Hutter & Ache, 2016; Soll & Larrick, 2009; Yaniv & Milyavsky, 2007). However, advice is more heavily weighted when the advisor is trusted by the decision-maker (Sniezek & Van Swol, 2001), when the advice is paid for (Gino, 2008), and when the task is difficult (Gino & Moore, 2007).

In the advice-taking literature, the extent to which individuals use advice is often evaluated using the “Judge-Advisor System” (JAS; Sniezek & Buckley, 1995). In the JAS, the experimental procedure involves an estimation or a choice task. The judge is asked to provide an initial response. Then, they receive the advisor’s (or multiple advisors’) response and can revise their first response accordingly. This design enables inferring the weights that the judge assigns to their estimate and the advisor’s estimate. However, when the judge is always exposed to the problem before receiving (or choosing whether to receive) the advisors’ responses, it is impossible to examine whether people are willing to delegate the decision to others even without experiencing the problem by themselves. The WoC phenomenon implies that relying on the aggregated crowds’ decisions is often more reward-maximizing than individual decision making. However, it is currently unclear whether people realize it, and whether they will behave accordingly. In contrast to the JAS paradigm, the present study examined situations where the judge must choose between two mutually exclusive options: either rely on their estimate or only rely on the WoC. Although the latter was the rational decision in terms of reward-maximization, it is unclear whether participants would prefer this strategy or adopt a self-reliant, suboptimal approach.

People’s willingness to take or give advice is related to their confidence. For example, individuals with low self-confidence tend to seek and rely on advice (Gino, Brooks, & Schweitzer, 2012), and advisors adapt their confidence levels to the weight they want the judges to attribute to their advice (Ache, Rader, & Hütter, 2020). Moreover, participants’ confidence in their own decision is negatively related to their use of social information (Morgan, Rendell, Ehn, Hoppitt & Laland, 2012), and initial individual evaluations are especially affected by the opinions of highly confident group members (Moussaïd, Kämmer, Analytis & Neth, 2013). Based on this literature, we examined whether the mere *choice* to rely on the WoC is related to the participants’ confidence in their individual judgments.

In a series of five experiments, participants performed a random dot motion task (RDM). The experiment began with 10 practice trials, in which the participants had to decide whether the dots move to the right or the left. After each decision, they were given the distribution of left/right responses that was attributed to a group of other observers who viewed the same display. The practice trials aimed to familiarize participants with the perceptual decision and its difficulty, to familiarize them with the idea of observing the response distribution of a group of other observers, and to enable them to compare their decisions (right/left) to those of the group members presented in each trial. After a series of practice trials, a critical test trial appeared, in which they could earn an extra reward for a correct answer. To do so, they had to choose between observing the RDM display, or the distribution of group responses (but not both). Since individual-level accuracy in this task was above 50% (except for Experiment 2, see below), the probability that the majority decision among a group of independent observers was correct was larger than that of a single individual (Condorcet, 1785). Accordingly, relying on the WoC was the reward-maximizing choice in all the experiments. Nevertheless, our results show a strong and prevalent tendency to dismiss the WoC, which was associated with individuals’ overconfidence in their own estimates.

## 2. Experiment 1: Low RDM coherence, small group size

### 2.1 Method

**Figure 1:**
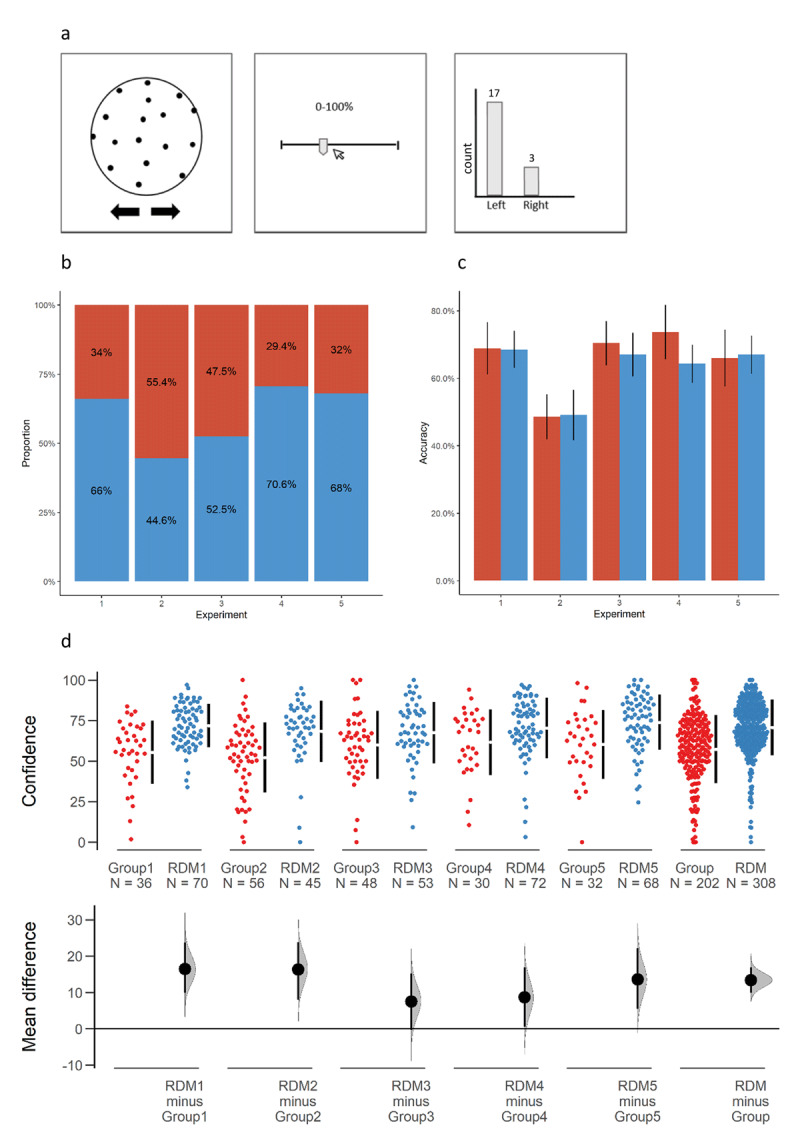
**(a)** A practice-phase trial in Experiment 1. **(b)** Distribution of choices in the test trial across all 5 experiments. **(c)** Practice-phase mean accuracy rates (±SE) in each experiment. **(d)** A multi two-group estimation plot (Ho et al., 2019). The scatter plot shows the observed confidence levels of each group in each experiment (and in all experiments combined in the rightmost plot) during the practice phase. The vertical lines beside each group indicate the mean ± 1 standard deviation. The effect size (mean difference) and 95% CI are positioned below the raw data. The curve indicates the resampled distribution of the mean difference, given the observed data.

The experiment was built using Qualtrics and was distributed to 107 participants (34 females, 72 males, mean age (years) = 36.6, sd = 10.7; Country: US – 73, India – 28, Brazil – 1, Italy – 3, Poland – 1) via Amazon Mechanical Turk (MTurk). One participant was excluded from the analysis due to an irrelevant answer in the final question of the experiment, referring to a (presumably different) experiment that required grammatical judgments. Accordingly, a total of 106 participants are included in the results. The experiment lasted 13 minutes on average. Each participant received a fixed payment of $2 and could earn an additional $2 depending on their accuracy in the test trial of the experiment (see below). The experiment and all subsequent experiments were approved by the Ethics Committee of the Department of Psychology at Ben-Gurion University of the Negev in accordance with American Psychological Association guidelines. The experiment was composed of a 10-trial “practice” phase, followed by a single “test” trial. In each of the practice trials participants performed a random dot motion (RDM) task (see ***Figure 1***). In this task, an array of 150 moving dots appeared on the screen within a borderless circle. Only 5% of the dots moved coherently to the right or the left side of the screen, while the other dots moved randomly. The participants had to indicate the movement direction, and their confidence in their response using a 0–100% analogue scale, in a self-paced manner. After submitting their responses, the participants passively viewed a bar graph showing the distribution of left/right responses that was attributed to a group of 20 other observers who performed that trial. The distributions of decisions were generated by drawing from a binomial distribution with N = 20 and a success probability (namely, an individual accuracy rate) of 67%. This accuracy rate was based on the results of a previous study (N = 43) conducted in our lab using the RDM task with similar visual parameters. Also, we only presented draws from the binomial distribution in which the majority decision was compatible with the correct motion direction. While feedback concerning the correct response was not provided, these decision distributions only provided the participants with information on whether their response aligns with the majority decision or not. In the test trial, participants were instructed to answer as accurately as possible and were given a monetary incentive to do so, as a correct response in this trial would earn them an additional $2. They were presented with the following choice: “You need to make a decision about the direction of movement of an array of dots. The dots can move either to the right or to the left. In order to make your decision, you can watch a video clip of the moving dots or the responses of 20 other participants who viewed this video, but not both”. Since the individual-level accuracy in the task was only 67%, and the probability that the majority among 20 participants is correct was 91.3% (calculated from a binomial distribution), the reward-maximizing decision in the test trial was to observe the group distribution rather than the RDM display.^1^ After observing the chosen stimuli in the test trial, the participants had to indicate their left/right decision and a 0–100% confidence. Also, they were asked to shortly explain the reasons behind their choice. To test the potential role of individual differences in the choice to use or dismiss the WoC, participants completed a short version of the Rational-Experiential Inventory (REI-10, Epstein et al., 1996) that assesses preferences for information processing style, a short version of the Narcissistic Personality Inventory (NPI-16, Ames et al., 2006), and a single self-criticism item (“I tend to be very self-critical”), scored on a scale from 1 (strongly agree) to 7 (strongly disagree). The Rational-Experiential Inventory is composed of two sub-scales – Need for Cognition (NFC) and Faith in Intuition (FI). Individuals higher in NFC are described as more rational decision-makers (Curşeu, 2006) and were also reported to actively seek advice and recognize others as valuable information sources (Curşeu, 2011). Individuals who score high on the FI scale rely more heavily on intuition and past experience and are often guided by reinforcement-based heuristics in their decisions (Alós-Ferrer & Hügelschäfer, 2012). Narcissistic tendencies are positively associated with overconfidence (Campbell et al., 2004) which is pertinent to the choice of whether or not to utilize external sources of information in decision making.

### 2.2 Results

A majority of 66% (70 out of 106 participants) chose to make their final decision by watching the RDM display by themselves. Reasons for this choice included “I preferred to judge for myself than to rely on others’ decisions”, “I didn’t always agree with what the majority thought”, “I felt confident in my ability” and “If I’m going to be right or wrong I want it to be because I chose the outcome” (see full data in *https://osf.io/ztj73/files/*).

Notably, participants who chose to watch the RDM display were more confident in their decisions than those who chose to see the distribution of group responses (see ***Table 1***). This was true already during the practice trials, *t*(104) = 5.140, *p* < .001, *Cohen’s d* = 1.054 (confidence averaged across all 10 practice trials), as well as in the test trial, *t*(104) = 3.262, *p* = 0.001, *Cohen’s d* = 0.669. However, no significant differences were found in the accuracy rates of the two groups in the practice trials, χ^2^(1) = 0.011, *p* = 0.916, *Cramer’s V* = 0.003. In the test trial, however, those who chose to watch the distribution of group responses were significantly more accurate compared to those who viewed the RDM display, χ^2^(1) = 4.448, *p* = 0.035, *Cramer’s V* = 0.205. This was expected as most of them (34 of 36, 94.4%) relied on the majority decision which pointed to the correct response.

**Table 1:** Accuracy rates and confidence ratings in practice and test trials for Experiments 1–5.


EXPERIMENT	DECISION	N	%	PRACTICE TRIALS		TEST TRIAL	

				CONFIDENCE (MEAN ± SD)	ACCURACY	CONFIDENCE (MEAN ± SD)	ACCURACY

1	RDM	70	66.0%	71.8 (±13.3)	68.6%	75.1 (±16.3)	78.6%

	Group	36	34.0%	55.3 (±19.4)	68.9%	63.5 (±19.0)	94.4%

2	RDM	45	44.6%	68.4 (±18.9)	49.1%*	78.3 (±19.3)	84.4%

	Group	56	55.4%	52.1 (±21.6)	48.6%*	57.2 (±23.7)	96.4%

3	RDM	53	52.5%	67.5 (±18.8)	67.0%	73.9 (±22.1)	84.9%

	Group	48	47.5%	59.9 (±20.9)	70.4%	67.7 (±20.2)	97.9%

4	RDM	72	70.6%	70.3 (±18.6)	64.3%		

	Group	30	29.4%	61.6 (±20.2)	73.7%		

5	RDM	68	68%	73.8 (±17.0)	67.0%	75.8 (±19.8)	67.6%

	Group	32	32%	60.2 (±21.2)	66.0%	65.9 (±21.5)	93.7%

Overall	RDM	263	64.3%	70.6 (±17.3)	66.7%**	75.7 (±19.2)	78.0%

	Group	146	35.7%	57.2 (±20.9)	69.7%**	63.1 (±21.7)	95.9%


* Accuracy here refers to congruence between participants’ decision and the group decision. ** Experiment 2 was excluded.

We moved to examine whether participants’ confidence ratings were calibrated with their accuracy and whether the participants who chose to watch the RDM stimuli differed in their calibration from those who chose to see the group distribution. Accordingly, we compared confidence ratings during the practice phase between trials in which the response was correct and those in which the response was incorrect. An ANOVA was conducted with Confidence as a dependent variable, Choice (RDM, Group) as a between-subject factor, and Correctness (correct vs. incorrect trials) as a within-subject factor (see ***Table 2***). The main effect of Correctness was significant, reflecting a higher confidence in correct trials. However, the two-way interaction was clearly non-significant, suggesting that the participant’s calibration was equivalent in the two choice groups (RDM, Group).

**Table 2:** Means and analysis of variance results for confidence judgments in practice trials.


EXPERIMENT	DECISION	N*	CONFIDENCE		MAIN EFFECT OF CORRECTNESS	CORRECTNESS*CHOICE INTERACTION

			CORRECT TRIALS (MEAN ± SD)	INCORRECT TRIALS (MEAN ± SD)		

1	RDM	69	73.3 (±12.9)	68.6 (±16.5)	*F*(1,103) = 27.75, *MSe* = 46.53,*p* < .001, η*_p_*^2^ = .21	*F*(1,103) = .27, *MSe* = 46.53,*p* = .602, η*_p_*^2^ = .003

	Group	36	57.1 (±19.0)	51.3 (±20.6)		

3	RDM	51	68.7 (±18.9)	64.1 (±22.2)	*F*(1,95) = 18.29, *MSe* = 71.59,*p* < .001, η*_p_*^2^ = .16	*F*(1,95) = .22, *MSe* = 71.59,*p* = .643, η*_p_*^2^ = .002

	Group	46	60.7 (±20.3)	54.9 (±22.4)		

4	RDM	70	71.7 (±17.5)	69.3 (±19.3)	*F*(1,97) = 9.41, *MSe* = 62.27,*p* = .003, η*_p_*^2^ = .09	*F*(1,97) = 1.14, *MSe* = 62.27, *p* = .287, η*_p_*^2^ = .012

	Group	29	62.2 (±20.8)	57.1 (±24.3)		

5	RDM	67	74.4 (±17.1)	72.2 (±20.6)	*F*(1,97) = 3.32, *MSe* = 44.65,*p* = .072, η*_p_*^2^ = .033	*F*(1,97) = .12, *MSe* = 44.65,*p* = .730, η*_p_*^2^ = .001

	Group	32	60.3 (±21.9)	58.8 (±20.7)		

Overall	RDM	257	72.3 (±16.6)	68.8 (±19.7)	*F*(1,398) = 53.63, *MSe* = 56.17,*p* < .001, η*_p_*^2^ = .119	*F*(1,398) = 1.26, *MSe* = 56.17,*p* = .262, η*_p_*^2^ = .003

	Group	143	60.0 (±20.3)	55.3 (±21.9)		


* Participants that were only correct, or only incorrect during all practice trials were removed from the analysis.

No significant group differences were observed in the NFC sub scale of the REI, *t*(104) = –0.435, *p* = 0.664, *Cohen’s d* = 0.089, *BF_10_* = 0.234, nor in FI, *t*(104) = 1.620, *p* = 0.108, *Cohen’s d* = 0.332, *BF_10_* = 0.683. No significant differences were observed in NPI either, *t*(104) = 1.254, *p* = 0.213, *Cohen’s d* = 0.257, *BF_10_* = 0.431. We posited that individuals higher in narcissism will report higher confidence in their decision, but this was not supported, *r* = 0.132, *t*(104) = 1.361, *p* = 0.176, *BF_10_* = 0.333. Also in contrast to our expectations, participants who chose to watch the RDM display scored significantly higher on the self-criticism item, mean = 4.24 vs. 3.33, respectively, *t*(104) = 2.642, *p* = 0.009, *d* = 0.542. While relatively little is known about individual differences in personality traits and their relationship to social learning (see Mesoudi, Chang, Dall & Thornton, 2016, for review and critical discussion), our null results may help exclude NFC, Narcissistic tendencies and self-criticism as potential contributing factors.

## 3. Experiment 2: Zero RDM coherence, small group size

Previous experimental work and theoretical considerations suggest that social information use is sensitive to task difficulty, individual-level performance, and individual confidence (Gino & Moore, 2007; Morgan et al., 2012; Toelch, Bruce, Newson, Richerson & Reader, 2014). Although the task of Experiment 1 was already considerably difficult (<70% accuracy on average), we hypothesized that further increasing the difficulty would increase the reliance on social information. This is expected both due to the effect of difficulty per se, and due to the reduction in confidence, that was expected to be associated. To test these ideas, we chose to use a situation in which the information given to individuals is completely random. This was done by presenting them with 0% coherence RDM displays. In such a case there is no meaning to the “correctness” of the individual responses. However, by presenting the same group distributions that were used in Experiment 1, we created a situation in which the *perceived correspondence* of the individual decisions to those of the group was at chance level. We predicted that this will reduce individual’s confidence in their own decisions, and as a result will increase reliance on the WoC.

### 3.1 Method

102 participants were recruited via MTurk (43 females, 58 males, 1 other, mean age (years) = 37.4, sd = 10.9; Country: US – 90, India – 9, Brazil – 1, Canada – 1). One participant was excluded from the analysis due to an irrelevant answer in the final question of the experiment, so a total of 101 participants are included in the results. The procedure was similar to that of Experiment 1 with a single modification – the RDM task involved a 0% coherence in the practice phase, namely a complete random motion. In such a situation the decision was based entirely on noise. We used the same distributions of group decisions as in the previous experiment, so no congruence was expected between the participants’ own left/right decisions and the group decisions. In the test trial, we used a 5% coherence RDM display rather than 0% so we could pay the bonus reward based on accuracy in this trial, as was done in Experiment 1. The REI-10 and NPI-16 questionnaires were omitted from this experiment in light of the results of Experiment 1, and only the self-criticism item was administered.

### 3.2 Results

55.4% (56 out of 101) of the participants preferred viewing the group responses in the test trial. This proportion was significantly lower than that observed in Experiment 1, χ^2^(1) = 9.576, *p* = 0.002, *Cramer’s V* = 0.214. Still, a substantial proportion of participants (44.1%) chose to adhere to their own judgment of the RDM display.

To examine whether our manipulation led to lower confidence compared to Experiment 1, we conducted a two-way ANOVA with mean Confidence over the 10 practice trials as a dependent variable, and Choice (RDM, Group) and Experiment (1 vs 2) as a between-subject factor. The main effect of Choice was significant, *F(1,203) = 39.71, MSe = 329.13, p < .001*, η*_p_^2^ = .163*, but not the main effect of Experiment, *F(1,203) = 1.65, MSe = 329.13, p = .20*, η*_p_^2^ = .008*, nor the 2-way interaction, *F(1,203) = 0.001, MSe = 329.13, p = .974*, η*_p_^2^ < .001*. Accordingly, the different choice proportions in the two experiments were not driven by lower confidence in Experiment 2.

As in Experiment 1, participants who chose to view the RDM display in the test trial were significantly more confident than those who chose to view the distribution of group responses. This was true both in the practice trials, *t*(99) = 3.979, *p* < .001, *Cohen’s d* = 0.796, and in the test trial, *t*(99) = 4.814, *p* < .001, *Cohen’s d* = 0.964.

There was no correct answer in the practice phase trials, and hence no accuracy. However, we tested whether there were group differences in the congruence between the individual decisions and the majority decision of the group distribution in those trials. This was done to rule out the possibility that the final choice was correlated with the perceived correctness in the practice phase. As expected, overall congruence between participants’ responses and group responses during the practice trials was not statistically different from chance level (49.3%, *p* = 0.682). Congruence during the practice phase did not differ between the groups, χ^2^(1) = 0.029, *p* = 0.864, *Cramer’s V* = 0.005. Since the test trial involved a 5% coherence stimulus, those who viewed the group responses were again more accurate, but not significantly so, χ^2^ = 4.415, *p* = 0.078 (computed by Monte Carlo simulation to deal with small expected counts), *Cramer’s V* = 0.209.

Unlike Experiment 1, no group differences were found in the self-criticism item, *t*(99) = –0.269, *p* = 0.788, *Cohen’s d* = 0.054, *BF_10_* = 0.218. Accordingly, this item was not used in the following experiments.

## 4. Experiment 3 – Low RDM coherence, large group size

Social information use and advice-taking behavior are largely affected by group size (King & Cowlishaw, 2007; Morgan et al., 2012). Information gathered by other individuals is more likely to be correct as more individuals are involved. This results in down-weighting individually-gained information as a function of advisors’ group size. This tendency is rational in situations when both the individual and the advisors are exposed to the same information. However, such a strategy was impossible to implement in our case, where participants could only gain from one source (self or others). Condorcet’s (1785) Jury Theorem holds that when individual accuracy is higher than chance, the group majority is more accurate than individual’s decision for *any group size*. According to this principle, observing the group response was the accuracy-maximizing decision in our task, regardless of the group size. Still, based on the general sensitivity of social learning to the number of demonstrators, we hypothesized that people might be more likely to rely on the WoC when it reflects the opinions of a larger group. The following experiment was identical to Experiment 1, except for providing the participants information gained from groups of 200 people rather than only 20.

### 4.1 Method

101 participants were recruited via MTurk (41 females, 60 males, mean age (years) = 38.9, sd = 12.61; Country: US – 87, India – 11, Canada – 1, Germany – 1, Pakistan – 1). The procedure of Experiment 1 was used with one modification. Here, the group distributions of left/right responses that were presented in the practice phase were attributed to a group of 200 observers and were generated by drawing from a binomial distribution with N = 200 and *p* = 0.67, in which the majority always indicated the correct decision. No questionnaires were administered to assess individual differences.

### 4.2 Results

52.5% (53 out of 101) of the participants chose to make their final decision based on watching the RDM display by themselves. As predicted, this proportion was significantly lower than in Experiment 1, χ^2^(1) = 4.158, *p* = 0.041, *Cramer’s V* = 0.141.

As in the previous experiments, higher confidence was observed among participants who chose to view the RDM display in the test trial compared to those who preferred to view the group responses. However, as opposed to the previous experiments, this pattern did not reach significance, neither in the practice phase, *t*(99) = 1.909, *p* = 0.059, *d* = 0.38, nor the test trial, *t*(99) = 1.465, *p* = 0.146, *d* = 0.292.

As in Experiment 1, participants were more confident in correct compared to incorrect trials during the practice phase, but this difference was similar for both participants who chose to watch the RDM display and those who chose to see the group distribution. Also, as before, no significant group differences were found in accuracy during the practice trials, χ^2^(1) = 1.38, *p* = 0.24, *Cramer’s V* = 0.037. In the test trial, those who viewed the group responses were again more accurate, χ^2^ = 5.253, *p* = 0.032 (computed by Monte Carlo simulation to deal with small expected counts), *Cramer’s V* = 0.228.

## 5. Experiment 4 – Advising another person

The previous experiments demonstrated a sub-optimal decision-making bias towards using individually gathered information. A possible reason for this bias is a correspondence between the origin of the information and the target of the decision, being the decision-maker. Namely, it could be that people choose to prefer their own judgment because they are the ones who are going to be influenced by the decision. Another factor that might have amplified this effect was the bonus given to correct answers in the test phase. The present experiment aimed to eliminate these possibilities by asking the participants to instruct others how to behave in the test trial, rather than by asking them to decide for themselves.

Choosing for others reduces pre-decisional biases (Polman, 2010) and increases information search (Liu, Polman, Liu & Jiao, 2014) compared to choosing for oneself. Accordingly, we seek to examine whether advising another person regarding the choice in the test phase would lead to a more reward-maximizing behavior compared to the previous experiments. The practice phase in the present experiment was identical to that of Experiment 1 and was followed by the instruction to advise another person how to perform the test phase. Contrary to our predictions, this manipulation did not affect the tendency to rely on self-gathered information.

### 5.1 Method

102 participants were recruited via MTurk (37 females, 64 males, 1 other, mean age (years) = 35.3, sd = 10.52; Country: US – 74, India – 22, Brazil – 2, Italy – 1, Philippines – 1, Other – 2). The practice phase was identical to that of Experiment 1. In the test trial, the following scenario was described:

*“Sarah is participating in an experiment similar to this one. She is asked to make a decision about the direction of movement of dots, just as you did before. Unlike you, she only needs to answer one question regarding one array of dots. In order to answer correctly, Sarah can either: (a) watch the video of the moving dots, or (b) see the responses of a group of 20 other people who viewed this video. However, she cannot see both. What would you advise Sarah to do in order to maximize her chances of answering correctly?”*.

Half of the participants were randomly assigned to read this scenario, while the other half read the same scenario about an individual named John. After indicating their answer, the participants were asked to briefly explain their choice of advice. Unlike the previous experiments, no test trial was administered.

### 5.2 Results

A majority of 70.6% (72 out of 102 participants) advised Sarah/John to watch the RDM display by themselves. This pattern did not differ significantly from the one observed in Experiment 1, χ^2^(1) = 0.433, *p* = 0.510, *Cramer’s V* = 0.046.

As in the previous experiments, participants whose advice was to view the RDM display were significantly more confident during the practice phase compared to those who advised viewing the distribution of group responses, *t*(100) = 2.099, *p* = 0.038, *Cohen’s d* = 0.456. As before, no differences in calibration were found among the groups (***Table 2***).

Accuracy rates during the practice phase were higher among participants who advised to watch the distribution of group responses, χ^2^(1) = 8.401, *p* = 0.004, *Cramer’s V* = 0.091. This difference in accuracy was not observed in the previous experiments, and hence should be taken with caution.

## 6. Experiment 5 – Low RDM coherence, small group size, with feedback

In the final experiment, we wished to address the possibility that the wide rejection of group information is linked to the lack of feedback during the practice trials. The previous experiments did not include feedback during the practice phase so that the choice in the test trial was not confounded by learning the correctness of the alternatives. If feedback was provided, we could not know whether people rely on the group responses due to the general properties of the WoC, or because they simply learned that this choice was always correct. In other words, choosing the WoC could simply reflect the fact that the group distribution was always aligned with correctness during the practice phase. However, a possible account for the findings of the previous experiments is that the lack of feedback during the practice phase, in combination with the relative difficulty of the decision, prevented the participants from knowing what the correct answer in each trial was. Accordingly, in situations where the group distribution pointed to an opposite response than the one given by themselves, they were unable to know that the group response, rather than their own, was correct. According to this account, giving feedback after each trial of the practice phase will mark the group response as correct, and may shift the participants’ tendency toward adhering to the WoC.

### 6.1 Method

100 participants were recruited via MTurk (34 females, 66 males, mean age (years) = 35.2, sd = 10.3; Country: US – 94, India – 2, Brazil – 4). The procedure of Experiment 1 was used with the addition of feedback during the practice trials. After making a decision and rating their confidence and before viewing the group responses, the participants were given the correct answer (“Correct/Wrong. The dots were moving to the left/right”). This presented the participants with two additional pieces of information compared to previous experiments: their level of performance on the task and the fact that the majority was always right. No questionnaires were administered to assess individual differences.

### 6.2 Results

A majority of 68% (68 out of 100 participants) chose to make their final decision based on watching the RDM display by themselves. This proportion was not significantly different than the one observed in Experiment 1, χ^2^(1) = 0.089, *p* = 0.765, *Cramer’s V* = 0.021. Accordingly, providing feedback did not alter the pattern of results observed in the above experiments. The participants’ tendency to favor their own judgment was unaffected by demonstrating the superior performance of the group.

Consistent with previous experiments, participants who chose to view the RDM display in the test trial were significantly more confident compared to those who preferred to view the group responses during the practice trials, *t*(98) = 3.437, *p* < .001, *d* = 0.736, as well as in the test trial, *t*(98) = 2.267, *p* = 0.025, *d* = 0.486.

No significant group differences were found in accuracy during the practice trials, χ^2^(1) = 0.091, *p* = 0.762, *Cramer’s V* = 0.01. As before, in the test trial, those who viewed the group responses were more accurate, χ^2^ = 8.128, *p* = 0.005 (computed by Monte Carlo simulation to deal with small expected counts), *Cramer’s V* = 0.285.

## 7. Discussion

The results of Experiments 1–5 consistently show that a substantial proportion of the participants preferred to make their final decision based on the RDM display rather than on the distribution of group responses. Moreover, this choice was related to higher confidence levels during the task, which did not reflect higher accuracy (see Yaniv, Choshen-Hillel & Milyavsky, 2009, for an additional dissociation between confidence and accuracy). While the participants’ confidence ratings were higher in correct than incorrect trials during the practice phase, this difference did not interact with the participants’ choice in the test trial. Accordingly, the choice in the test trial was unrelated to calibration in the practice trials.

The bias we observed in participants’ behavior is threefold. First, dismissing the WoC results in suboptimal accuracy. Despite being a valuable source of information, individuals waived the opportunity to use the WoC. Research using the JAS paradigm demonstrated that people tend to down weight advisors’ judgment and privilege their own opinions (Yaniv, 2004; Yaniv & Kleinberger, 2000). Typically, however, these studies present participants with the answer of one advisor only. Accordingly, the degree to which people combine their own opinion with that of a single advisor does not inform us regarding the adoption or dismissal of the WoC by individual decision-makers. Moreover, when only one advisor is presented, the choice to discount their opinion, and in some cases to even completely ignore it, is not necessarily irrational. By contrast, in the present study, we contrasted individual decision-making with total reliance on the WoC. Our results demonstrate an irrational (or, non-reward-maximizing) bias, by which people choose the less accurate decision strategy.

One explanation for this seemingly suboptimal strategy is the minimization of potential conflict. Sharot & Sunstein (2020) suggest that for information-seeking to initiate, people must predict the content of the information and estimate its value in terms of its instrumental utility (namely, the potential to increase reward and avoid harm by using this information), hedonic utility (namely, will this information induce negative or positive affect) and cognitive utility (namely, the power of the information to instill a better understanding and anticipation of reality). The result of these combined estimates can either trigger information-seeking or lead to its avoidance. The process of predicting the value of information is highly susceptible to biases, one of which is overconfidence.

Overconfidence is a robust finding in psychology and may take many forms, by which individuals often overestimate their actual ability and perceive themselves to be better than average. For example, individuals consider themselves to be more skilled than the average driver (Svenson, 1981), new business owners are extremely optimistic regarding the likelihood of their business to succeed (Cooper et al., 1988), and people even associate themselves with more fair behaviors compared to others (Messick et al., 1985). Our results indicate that overconfidence, as demonstrated in the responses during the practice phase, was related to over-reliance on personal information and a broad dismissal of the WoC.

Boosting accuracy by accepting advice comes with the price of compromising decision autonomy. People generally value their autonomy and tend to adhere to their own opinion (Baer & Brown, 2012, but see Kassirer et al., 2020). Accordingly, they have a sense of psychological ownership towards decisions or ideas that they develop on their own, and revising of opinions in response to environmental feedback will take place more often when the feedback adds to this sense of ownership. This ties directly to the notion of avoidance of information which has a potentially negative, or conflicting utility in the form of loss of agency and control over the final decision. It may be the case that observing the RDM actually maximizes participants’ utility.

A second bias observed in our results is stronger reliance on the WoC when it reflects a larger group (20 vs. 200 members; Experiments 1 vs. 3). Indeed, given an individual accuracy rate of 68% (calculated from participants’ performance in experiments 1, 3, and 4), the probability that the majority will be correct is larger for a group of 200 individuals (99.99%) than for a group of 20 individuals (92.8 %). However, in both cases, by using the WoC one has a better likelihood of being correct.

Lastly, the third bias is exhibiting sensitivity to perceived individual performance. In Experiment 2, the stimulus conveyed no information, and participants were unable to rely on their own judgment. This is reflected by the inconsistent feedback they received from observing the group distributions during the practice phase. Furthermore, any sense of confidence was spurious and had no anchor in actual performance. In this instance of complete uncertainty, there was a slight preference to use the group information. Still, the proportion of people who chose to rely on themselves in this experiment was substantial, as well as associated with overconfidence, as in the other experiments.

A possible explanation for our findings is that most of the participants chose not to use the group information because they were not told anything about the nature of this group. As a result, they were perhaps inclined not to trust the group or even suspect that the information was made up or somehow manipulated. However, this explanation is very unlikely. First, when examining participants written reports regarding the reasons for choosing to make a self-based decision, only a handful made explicit references about the possibility that the group information is fictitious, and another handful explicitly mentioned characteristics of the group as the reason for distrust (for instance, “I don’t trust the decisions of others I don’t know”). Moreover, when feedback was provided (Experiment 5), which revealed the fact that the majority decision always aligned with the correct response, the dominant decision among participants was still self-based. More importantly, the choice to make a self-based decision was associated with higher confidence already at the study phase, in which the information regarding the group responses was irrelevant. This, together with the fact that there was a decrease in this choice when the individual and group responses were uncorrelated, makes it very unlikely that the dismissal of the group information can be attributed to participant’s lack of knowledge about the composition of the group alone. Indeed, some participants reported they did not trust the group (and mostly that they trusted themselves better), but this distrust is not necessarily due to lack of information about the group, but rather may reflect their own overconfidence.

Another possible account for our results is that watching the video was more enjoyable and interesting than observing the group distribution at the test phase. Although we cannot rule out this possibility, it is unlikely for two reasons. First, the participants could earn $2 for responding correctly in the test phase, in addition to the $2 they earned in the practice phase. In other words, the test phase enabled them to double their earnings. Given that these are MTurk workers, the assumption is that they are eager to maximize monetary reward, and hence their decision at the test phase reflects their view of what an optimal decision is, rather than other factors such as fun and interest. Second, the above account cannot explain the fact that participants’ choice was related to confidence, and the modulation of our effect by factors such as group size and task difficulty, rendering it very unlikely. Another possible account for the bias toward individual decisions is that the participants might have suspected (incorrectly) that they might be deceived during the test phase by the presentation of a group distribution that points at the wrong response. Although we cannot rule out this possibility, it is incompatible with the effects of confidence and moderating factors observed in our study.

To conclude, this new manifestation of overconfidence demonstrates how cognitive biases can lead to suboptimal use of social information. While the present study demonstrated the phenomenon and its relationship to overconfidence, further work is needed to establish better theoretical grounding and to examine how these biases can be mitigated.

## Data Accessibility Statement

Experiments 1–4 were pre-registered through OSF. These pre-registrations, as well as the full data, hypotheses, R scripts and links to the experiments on Qualtrics are available at the OSF website (*https://osf.io/ztj73/*). Experiment 5 was not pre-registered. In this manuscript and the accompanied repositories we include all the data, and report all data exclusions, all manipulations, and all measures in the study (Simmons, Nelson & Simonsohn, 2012).

## Footnotes

Since we only presented group distributions that were compatible with the correct response, this probability was in fact 100% within the experiment. However, since the participants were obviously not aware of our design decision, the probability of 91.3% refers to the likelihood of the group being correct *in principle*, given this task.
